# Editorial

**Published:** 1846-12

**Authors:** 


### THE MEDICAL EXAMINER.

### PHILADELPHIA, DECEMBER, 1846.

### MEDICAL CLASSES IN PHILADELPHIA.

In our last number, we gave it as our opinion that the number of medical Students in Philadelphia the present season, would be at least equal to that of last winter. That opinion is now confirmed—in fact, we have no doubt that the number is greater, and may be estimated, in round numbers, at from *ten to eleven hundred.* The class at the Jefferson College is about *five hundred*, and we have no doubt that there are from five to six hundred distributed among the other schools.

### ECTROTIC TREATMENT OF SMALL-POX.

Our readers will remember, that in the August number of the *Examiner* we published some observations on the "Ectrotic treatment of small-pox by tincture of Iodine," from the pen of our much respected townsman, Dr. Samuel Jackson, late of Northumberland. In the last number of the "British American Journal of Medical and Physical Science," published at Montreal, (to the well stored pages of which we are frequently indebted for valuable articles, published in our Record,) the article is copied, with a claim of priority in that mode of treatment for Dr. Crawford, of Montreal. Dr. Crawford's paper is republished by our contemporary, and bears date at Montreal, March 15, 1844, and of course takes precedence of the published observations of our townsman; nevertheless, no one who knows Dr. Jackson will suppose for a moment that he had the least knowledge that Dr. Crawford or any one else had preceded him, or he would have taken pleasure in awarding to him the fullest credit. Dr. Dunglison, through whom Dr. Jackson's paper came to us, had seen the "Montreal Gazette," and made a note of Dr. C.'s observations, but did not deem it necessary to mention the circumstance. That no intention, however, existed on his part to withhold from our Canadian brother the credit due to him, is apparent from the fact, that his paper is expressly referred to and the date given, in the last edition of Dr. D.'s "New Remedies," under the head of "Iodinum," page 491, as follows: "Dr. Crawford, of Montreal, tried the comparative merits of tincture of iodine, and nitrate of silver, (in variola,) and gives the preference to the former. He found the application very manageable and very bearable." Having shown our contemporary's remarks to Dr. Jackson, he has sent us the following Card, which we have much pleasure in publishing: and in order to do full justice to Dr. Crawford, as well as for the sake of the valuable remarks which it contains, we have likewise transferred his paper to our "*Record.*"

"Dr. Jackson begs leave to state, that he never saw the 'Montreal Medical Gazette,' and that he never heard of it till to-day; that it was never known to one of the learned editors of this city; that in April, 1845, he took Drs. Nancrede and Bond to see his case of small-pox aborted by tincture of iodine; that neither of these, nor one of many others to whom he mentioned the subject, had heard of this medication; that he proposed to several physicians to repeat the experiment, which they did not, except Drs. Goddard and Sargeant; that for himself, he saw during the late epidemic only a few cases of mild varioloid, in which it was not important to experiment; that he is surprised to find, that of all the numerous periodicals of America and England, not one, as he believes, has noticed Dr. Crawford's experiments; surprised too that the editors of the British American Journal, his fellow citizens, should have withheld this courtesy; that though the small-pox has prevailed as an epidemic in New York, Baltimore, and Philadelphia, since the publication of Dr. Crawford, and every ectrotic was tried, no intimation of Dr. Crawford's paper got abroad in these places; that hence he has reason to hope that the editors of the British American Journal will not accuse him of appropriating Dr. C.'s labours; that he, Dr. Jackson, most cheerfully accords the priority of the experiment to Dr. C., on the authority of the British American Journal; that he is thankful to Dr. C. for having made more decisive experiments than his own; that he should not have published his solitary case, had not Professor Dunglison requested him to do so, that he might have it to refer to in the fifth edition of his '*New Remedies*,' which was then in the press; that some time after he had given his paper to Dr. Dunglison, and after it was printed, the Dr. spoke of Dr. Crawford's experiments, but he had lost the journal in which they were printed, having merely retained a memorandum; that the experiments of Dr. C. could not have made a strong impression on Dr. Dunglison, for he neither practised them in our late epidemic nor taught them to others; that he, Dr. Dunglison, never heard of Dr. C.'s experiments till his own was printed; that even Dr. Dunglison never saw more than one number of the Montreal Medical Gazette, and that the omnivorous editor of the American Journal of the Medical Sciences says, 'I saw it mentioned in a Boston paper, and this is all that I ever heard of it.' '*O curas hominum, O quantum est in rebus inane!!*'"

INSENSIBILITY DURING SURGICAL OPERATIONS PRODUCED BY INHALATION.

A certain Dr. Morton, a practising dentist in Boston, is advertising in the newspapers of this city, that he has secured a *patent* for what he calls "his improvement, whereby pain may be prevented in dentistical and surgical operations," and he now offers to sell "licenses to use said improvement," to "dentists, surgeons, and other suitable persons." Looking upon this as nothing more nor less than a new scheme to tax the pockets of the "enlightened public," we should not consider it entitled to the least notice, but that we perceive by the Boston Medical and Surgical Journal, that prominent members of the profession in that city have been caught in its meshes.

From a paper by Dr. H. J. Bigelow, "one of the Surgeons of the Massachusetts General Hospital," contained in the Boston Journal of the 18th of November, 1846, we derive the astounding information that Dr. Warren and Dr. Hayward—men at the very top of our profession—have allowed Morton to administer his "preparation"—"a secret remedy" for which he has taken out a patent—to patients on whom they were about to operate! Dr. Bigelow says, in extenuation of the course pursued by Morton in taking out a patent, that "it is capable of abuse, and can readily be applied to nefarious ends;" that "its action is not yet thoroughly understood, and its use should be restricted to responsible persons;" and that, one of its greatest fields is the mechanical art of dentistry, many of whose processes are, by convention, secret, or protected by patent rights. It is especially with reference to this art, that the patent has been secured."

Now we would like to know of Dr. Bigelow, whether any such restricted object is contained in the patent? None such appears in the proprietor's advertisement, and we apprehend that time will show that the sale is only limited by the price and disposition to purchase.

"We understand," says Dr. B. "already, that the proprietor has ceded its use to the Massachusetts General Hospital, and *that his intentions are extremely liberal with regard to the medical profession generally.*" Not a word of the sort is in the proprietor's advertisement. Did not Swaim give his *panacea* to the poor gratis, and a lot of ground to build a church on to boot? And did not John Williams, the oculist, with a trunk full of seals and royal testimonials, invite all the reverend clergy to come to him, and to bring with them all the poor blind people of their parishes, that he might cure them without money and without price?

The "*preparation* is inhaled from "a small two-necked glass globe," and smells of ether, and is, we have little doubt, an ethereal solution of some narcotic substance. The patient is rendered insensible for a period of from five or ten minutes to an hour; the pupils are dilated; "very young subjects are affected with nausea and vomiting, and for this reason Dr. M. has refused to administer it to children." In one case, a patient of Dr. Dix, "the respiration was very slow, the hands cold, and the patient insensible." Various active mesasures were found necessary to restore the patient, and, "complete consciousness returned only at the expiration of an hour."

We are persuaded that the surgeons of Philadelphia will not be seduced from the high professional path of duty, into the quagmire of quackery by this will-o'-the-wisp; and if any of our respectable dentists should be tempted to try this new "*patent medicine*," we advise them to consider how great must be the influence of an agent over the nervous system, to render a person unconscious of pain,—the danger there must necessarily be from such overpowering medication, and that if a fatal result should happen to one of their patients, what would be the effect upon their conscience, their reputation and business, and how the practice would be likely to be viewed by a Philadelphia court and jury? We cannot close these remarks, without again expressing our deep mortification and regret, that the eminent men, who have so long adorned the profession in Boston, should have consented for a moment to set so bad an example to their younger brethren as we conceive them to have done in this instance. If such things are to be sanctioned by the profession, there is little need of reform conventions, or any other efforts to elevate the professional character—physicians and quacks will soon constitute one fraternity.

---

### GUN COTTON.

The great topic of the day,—the theme of discussion every where, in Europe and America,—is the discovery of a process by which cotton is rendered as explosive as gunpowder. Various samples of cotton thus prepared, which we have seen, ignited with even more rapidity than the best gunpowder, and left not a trace behind when exploded on a piece of perfectly white paper. Speculation is of course rife as to the value of this new discovery—as to the safety of the article, its durability and economy, compared with gunpowder.

From all we can learn, we incline to doubt whether the discovery will prove of any great practical utility. It is alleged by a writer in one of the journals, "that it is constantly (though gradually) undergoing decomposition, and thereby deteriorating in quality," and it has been found to explode in the process of drying from spontaneous combustion. Cotton, it is known, is eminently hygrometric, and, in a moist atmosphere, from the absorption of water, it would be likely either to undergo decomposition, or lose its capability of ready ignition, and in either way be rendered unfit for use. It is also alleged by those who have given attention to the subject, that the cost of the manufucture of the new article, notwithstanding the facility of the process, will render it more expensive than gunpowder.

A late number of the London Medical Gazette contains the fullest account of its discovery, process of manufacture, etc., that we have any where seen, and we have accordingly transferred it to our *Record.* Experiments made in this city, have shown that the addition of a moderate quantity of sulphuric acid to the nitric, in the process of Dr. Otto, described in the extract, produces a more perfect article, but that spontaneous combustion is more apt to occur in drying.

